# Rapid onset of action and reduced nasal hyperreactivity: new targets in allergic rhinitis management

**DOI:** 10.1186/s13601-018-0210-2

**Published:** 2018-06-25

**Authors:** C. Bachert, J. Bousquet, P. Hellings

**Affiliations:** 1 Ghent University Hospital, Ghent, Belgium; 2 Upper Airways Research Laboratory, University of Ghent, Ghent, Belgium; 3 Karolinska Institute, Stockholm, Sweden; 4 Fondation FMC VIA-LR, Montpellier, France; 5 UMR-S 1168, INSERM U 1168, VIMA: Ageing and Chronic Diseases Epidemiological and Public Health Approaches, Villejuif, Université Versailles St-Quentin-en-Yvelines, Montigny le Bretonneux, France; 6 European Forum for Research and Education in Allergy & airways diseases (EUFOREA), Brussels, Belgium; 7 Laboratory of Clinical Immunology, Department of Microbiology and Immunology, KU Leuven, Herestraat 49, Box 1030, 3000 Louvain, Belgium; 8 Clinical Division of Otorhinolaryngology, Head and Neck Surgery, University Hospitals Leuven, Louvain, Belgium; 9 Clinical Division of Otorhinolaryngology, Head and Neck Surgery, Academic Medical Center, Amsterdam, The Netherlands

**Keywords:** Allergic rhinitis, MP-AzeFlu, Nasal hyperreactivity, Onset of action, Real life

## Abstract

**Background:**

This article summarizes a EUFOREA symposium, presented during the European Rhinology Research Forum in Brussels (9–10 November 2017; https://www.rhinologyresearch.eu/) which focused on novel pathways and therapeutic approaches in allergic rhinitis (AR).

**Main body:**

AR remains under-diagnosed, under-estimated and under-treated. A key component in understanding the AR landscape has been the realization of a significant mismatch between how physicians instruct AR patients to manage their disease and what AR patients actually do in real life. Data from the *Allergy Diary* (developed by MACVIA ARIA) showed that AR patients take their medication prn, rapidly switch treatments, often experience poor control, use multiple therapies and stop treatment when symptoms are controlled. Better control of AR may be achievable by using an AR treatment which has a rapid onset of action and which effectively targets breakthrough symptoms. Indeed, AR patients report complete symptom relief, lack of breakthrough symptoms, rapid onset of action, safety and use on an ‘as needed’ basis as key targets for new nasal sprays. MP-AzeFlu comprises intranasal azelastine and fluticasone propionate (FP) in a novel formulation delivered in a single device. It is the first AR treatment to break the 5 min onset of action threshold and provides clinically relevant symptom relief in 15 min, much faster than that noted for FP + oral loratadine. MP-AzeFlu also significantly reduces nasal hyperresponsiveness (NHR) which may be responsible for the breakthrough symptoms frequently reported by AR patients. Mechanisms underlying MP-AzeFlu’s effect include inhibition of mast cell degranulation, stabilization of the mucosal barrier, synergistic inhibition of inflammatory cell recruitment and a unique desensitization of sensory neurons expressing the transient receptor potential A1 and V1 channels.

**Conclusion:**

With the most rapid onset of action and onset of clinically-relevant effect of any AR medication currently available, and proven efficacy in the treatment of NHR, MP-AzeFlu is an AR treatment which provides what patients want, and fits how patients manage their AR in real life.

## Background

The European forum for research and education in allergy and airways diseases (EUFOREA; http://www.euforea.eu/) is an international non-profit organisation, forming an alliance of all stakeholders, from national and international organizations, institutions, and agencies working towards a common aim. The aim of EUFOREA is to reduce preventable and avoidable burden of morbidity and disability due to chronic airway diseases using personalized medicine, so that affected populations reach the highest attainable standards of health and productivity at every age, with these diseases no longer representing a barrier to patients’ well-being or socioeconomic development [1].

Allergic rhinitis (AR) represents the most common chronic airway disease in the world, affecting some 500 million individuals [2]. When symptomatic, AR sufferers experience a significant impairment of their quality of life [3], with negative effects apparent in all areas of daily living [4], and importantly impaired school performance [5] and work productivity [6, 7]. AR is a significant risk factor for the development of asthma [8], commonly occurs with asthma and affects asthma control to the same degree as smoking [2, 9–11]. The socioeconomic burden of AR in Europe is high, due mostly to its impact on work productivity [12]. Treatment of AR has been well identified [13] and algorithms are available [14]. Precision medicine is an important approach [15].

The second European Rhinology Research Forum held in Brussels (9–10 November 2017; https://www.rhinologyresearch.eu/), under the auspices of EUFOREA [16, 17] and the European Rhinologic Society, sought to explore research needs and priorities in upper airway diseases. A EUFOREA symposium presented during this meeting focused on novel pathways and therapeutic approaches in AR. The aim of this article is to summarize this symposium, and to discuss how the novel data presented may impact the management of AR.

## What patients with AR want

The aim of AR management is to gain control of the disease. As for asthma, the control concept is important for the rhinitis management landscape [18, 19]. Unfortunately, many patients with AR continue to live with uncontrolled disease [20]. In order to understand how AR control may be achieved in real-life, it is necessary to understand the complexity of the AR management landscape, and to know what AR patients really expect from their treatment.

Complexity of the AR landscape manifests in many ways. Firstly, a disparity exists in perspectives between physicians and AR patients, to the effect that AR is under-diagnosed, under-estimated and under-treated; the majority of physicians do not use adequate AR treatment [21]. Secondly, AR patient behaviour, as it pertains to their AR management, can be disorganized and inconsistent [22–24]. Up to two-thirds of AR patients simply forget to take their medication [24]. Thirdly, there is a reticence to seek professional medical advice, and a tendency to do so only when symptoms become ‘intolerable^’^ [22] or if symptoms persist after trying several over the counter (OTC) options [23]. An internet and telephone survey conducted with 2966 randomly selected adults with allergies showed that 52.6% of respondents had not seen a medical professional in the past year for their rhinitis symptoms, with some 30.2% preferring non-prescription medication because it did not involve a doctor visit [22]. Fourthly, AR patients glean a lot of information about their disease from the internet, and there is a high degree of self-management, a consequence of the large choice of OTC AR medications available [25–28]. It is therefore, crucial that AR care pathways should consider pharmacists [29]. The vast majority of AR patients use 2 or more AR medications in an effort to achieve faster and more effective relief from their nasal and ocular symptoms [30] (a practice which is not endorsed by the evidence) [31–33], with many patients calling for more efficacious OTC AR medications to better manage their AR symptoms [23]. Finally, most patients suffer from several episodes of short-term AR symptoms [30], and so rarely take their medication continuously for 14 days, as envisaged in the vast majority of seasonal AR randomized controlled trials (RCTs). Indeed, there is little evidence supporting the daily use of rhinitis treatment. For example, a recent study of pollen-AR in children found that on-demand fluticasone propionate (FP) treatment was at least as effective as a regular treatment at a lower dose [34].

To gain a better understanding of what patients want from their AR treatment, we should simply ask them. A survey conducted in Belgium showed that patients have high expectations from their AR treatment and have preferences in terms of AR medication delivery and approach [35]. 40% of these patients expected their AR to be cured by pharmacological intervention, with 30% preferring intranasal sprays (vs 24% for oral therapy), and 31% of respondents preferring a combination treatment with a step down approach. When asked, AR patients listed good symptom relief, lack of breakthrough symptoms, rapid onset of action, safety and use on an ‘as needed’ basis as key targets for new nasal sprays [23]. A different type of patient survey, called a discrete choice experiment quantified what patients want from their AR treatment, using a willingness to pay metric, and confirmed the results of the AR patient surveys [23, 35, 36]. The findings showed that patients want more complete symptom relief from their AR treatment and are willing to pay £43.81 (approx. €55) to achieve that (vs mild relief). Rapidity of response was also very important to AR patients, both in terms of time to maximum response and time to first response (i.e. onset). Patients were willing to pay £0.98 (approx. €1.10) for each hour faster to onset and £0.62 (approx. €0.70) for each day faster to maximum complete relief from AR symptoms. These surveys highlight the importance of involving patients in AR management and tailoring the AR treatment to the individual. By listening to AR patients we can empower them to establish their own treatment goals, manage treatment expectations, and incorporate treatment preferences into their AR treatment regimen. And this has benefits for the physician too; up to 96% of physicians consider that taking AR patients’ opinions into account increases therapeutic adherence [37]. Patient participation in the design of the treatment strategy is one of the 4 key pillars of precision medicine and believed to be a key success factor of treatment [17].

**Fig. 1:**
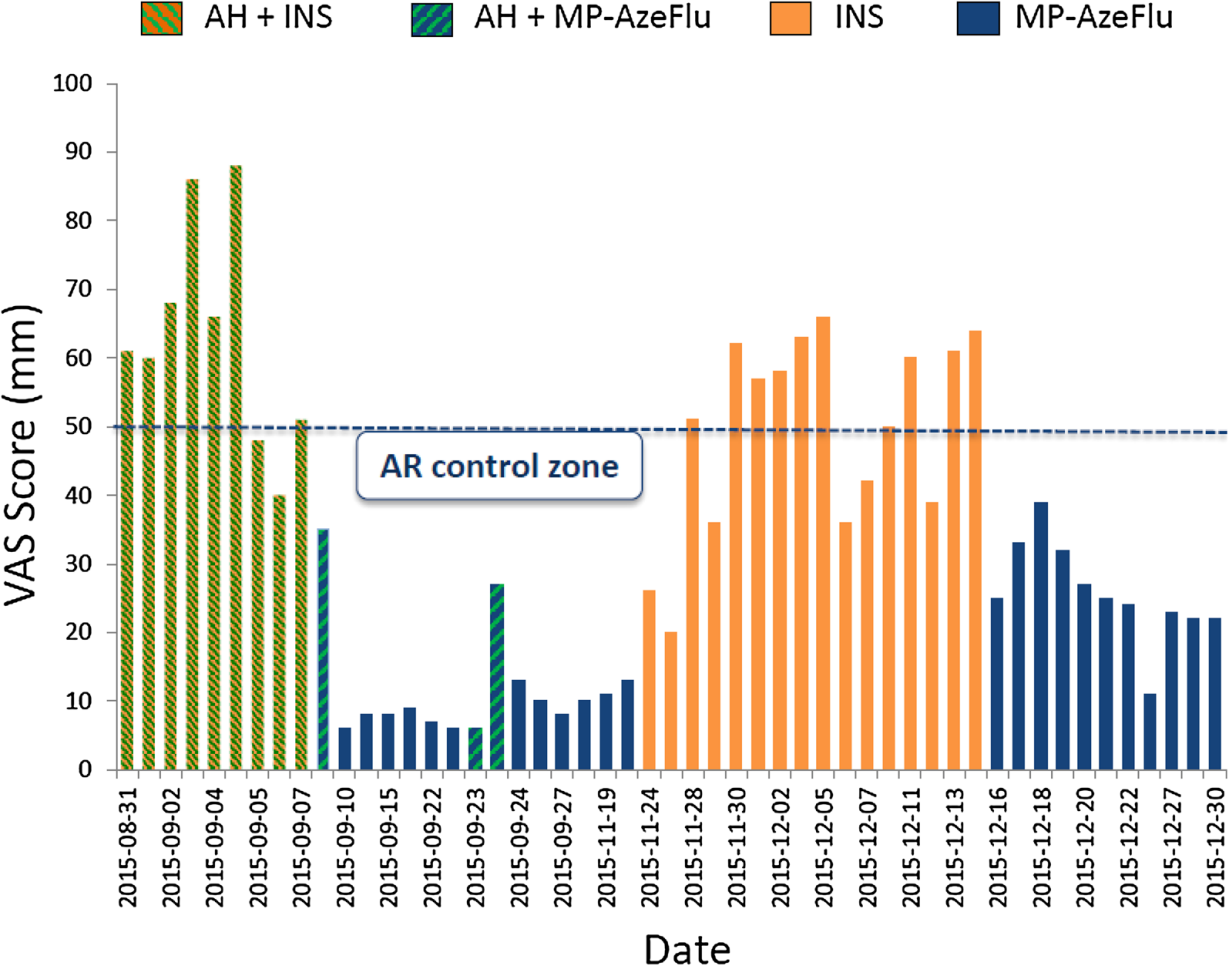
Allergic rhinitis (AR) control according to treatment in a single *Allergy Diary* user. VAS: visual analogue scale; AH: anti-histamine; INS: intranasal corticosteroid; MP-AzeFlu (MP-azelastine/fluticasone propionate); AR: allergic rhinitis. Reprinted with permission from MACVIA-ARIA

MACVIA-ARIA (Contre les Maladies Chroniques pour un Vieillissment Actif-Allergic Rhinitis and its Impact on Asthma) have recommended a patient-centered, mobile-health and clinical decision support system (CDSS) approach to achieve AR control in real life, linking all stakeholders in AR management (i.e. patients, healthcare providers (HCPs), regulators and guideline producers/implementers) [38]. This has been done by establishing a common language of AR control, a simple visual analogue scale (VAS) [18, 19, 39], and incorporating this VAS into free apps for patients [7, 40–42] and HCPs, as well as into an AR CDSS [18]. A VAS score cut-off of 5/10 cm is used to assess control and guide treatment decisions [18]. *Allergy Diary* is the app for patients. It is part of the MASK initiative (Mobile Airways Sentinel networK), and is included in the B3 action plan of the European Innovation Partnership on Active and Health Aging [43]. Data obtained from the *Allergy Diary* have enabled us for the first time to see how patients really treat their AR in real life and to recognize how this vastly differs from the RCT environment. The *Allergy Diary* data showed that In real-life patients (1) are poorly compliant with their AR treatment; (2) rapidly switch from one AR treatment to another if the desired response is not achieved; (3) often use multiple AR therapies; (4) fail to achieve sustained AR control on an intranasal corticosteroid (INS) monotherapy or anti-histamine + INS therapy; (5) achieve good control on more effective therapies, like MP-AzeFlu (Dymista^®^, Mylan Inc, USA); but (5) often switch back to less effective therapies when AR is well-controlled. Figure 1 shows *Allergy Diary* data from one typical AR user,inputted between Aug and Dec 2015. As can be seen, treatment is neither consistent nor consecutive. This user started with a multiple treatment regimen (INS + anti-histamine), switching to MP-AzeFlu (or MP-AzeFlu + anti-histmanine) to achieve MACVIA-ARIA defined disease control (i.e. VAS score < 5/10 cm). AR control was lost upon swiching to INS monotherapy, and regained once again on MP-AzeFlu (Fig. 1).

Better AR control may be achieved by listening to patients, taking account of how they actually manage their AR and providing what they want. Based on the evidence presented here, patients want an AR therapy, which provides complete and long-lasting symptom relief (i.e. no breakthrouh symptoms) and which has a rapid onset of action.

## Speed of onset of rhinitis treatment

MP-AzeFlu, is a relatively new addition to the AR treatment armamentarium, comprising intranasal azelastine (AZE) and FP in a novel formulation delivered in a single device [44]. Its onset of action has recently been investigated in an allergen exposure chamber and compared with an AR treatment regimen frequently used by AR patients in real life (i.e. anti-histamine + INS), in a single-centre, double-blind, double-dummy 3-period cross-over RCT [45]. AR symptoms were induced in asymptomatic patients (n = 82) in response to ragweed pollen challenge in the Ontario environmental exposure chamber. Patients then received a single dose of MP-AzeFlu (137/50 μg, 1 spray/nostril); intranasal FP (50 μg, 1 spray/nostril) plus oral loratadine (LOR; 10 mg) or placebo, and were monitored for 4 h. Symptoms were assessed at 5, 10, 15, 30, 60, 90, 120, 150, 180, 210 and 240 min after dosing. The primary outcome was onset of action measured by total nasal symptom score (TNSS). The TNSS comprises individual scores for nasal congestion, itching, rhinorrhoea and sneezing, with each of these scored from 0 (none) to 3 (severe), giving a maximum score of 12. Clinical relevance was assessed both from the individual and population perspective, using Kaplan–meier curves for time to response and minimally important clinical difference (MICD), respectively. A clinically relevant response was defined as at least 50% change from baseline in TNSS. MICD for TNSS was calculated using a sample size adjusted pooled estimate of baseline standard deviations. A difference of 0.47 points was defined as a small effect and 1.17 points defined as a medium effect [45].

**Fig. 2:**
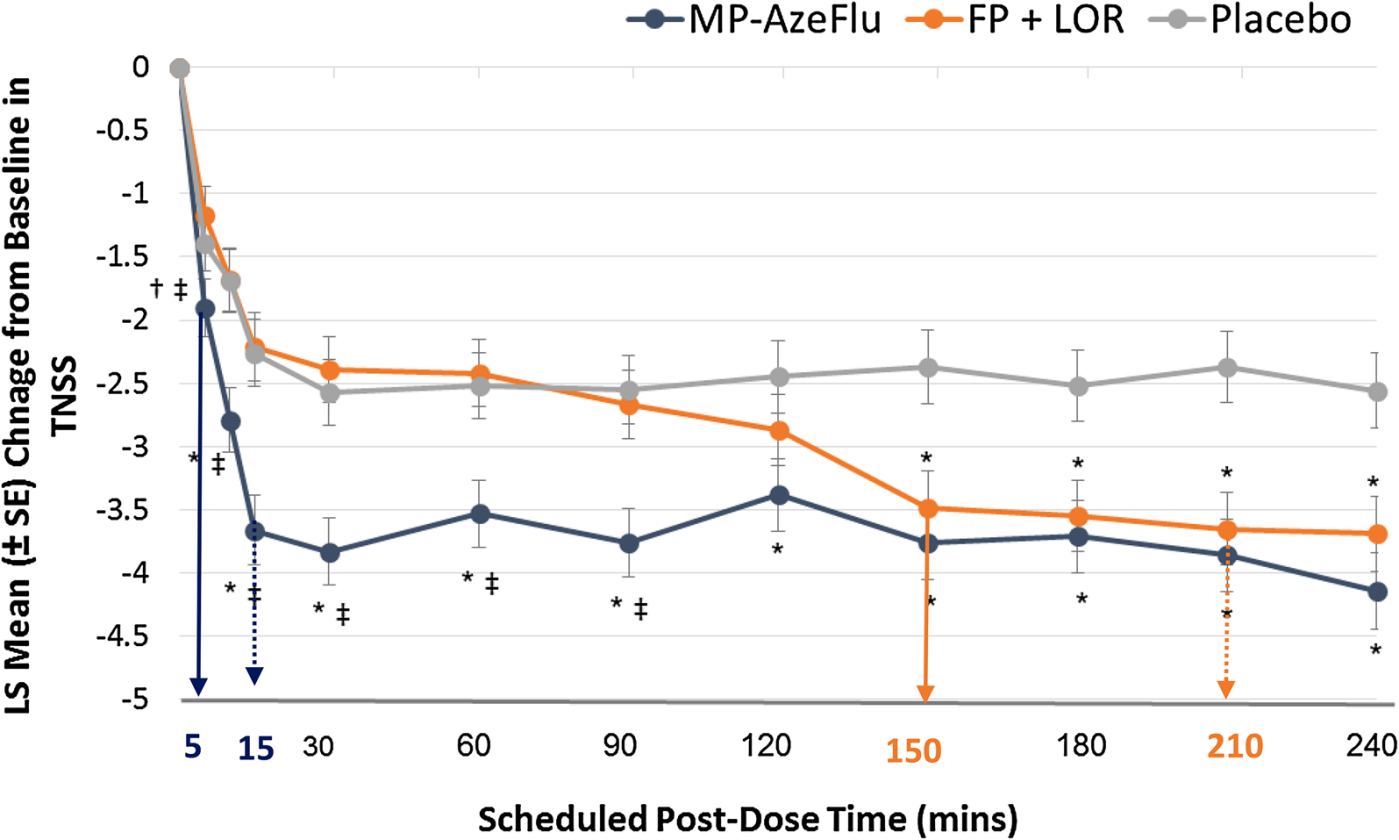
Effect of MP-AzeFlu, FP + LOR and placebo on nasal symptoms. Data are presented as mean change from baseline in total nasal symptom score (TNSS) assessed over a period of 4 h following exposure to ragweed pollen in an allergen exposure chamber. Arrow: onset of action; dotted arrow: onset to clinically relevant effect compared to placebo (i.e. 1.17 change in TNSS). MP-AzeFlu (MP-azelastine/fluticasone propionate; Dymista^®^; 1 spray/nostril; 138 μg/50 μg); FP (fluticasone propionate; Flonase^®^; 1 spray/nostril; 50 μg) + LOR (Loratadine; Claritin^®^; 10 mg). LS: least squares; SE: standard error. *p ≤ 0.005 vs placebo; ^†^p = 0.038 vs placebo; ^‡^p ≤ 0.003 vs FP + LOR. Modified from Bousquet et al. 2017 [45]

As can be seen from Fig. 2, the onset of action for MP-AzeFlu was 5 min compared to 150 min for FP + oral LOR, a difference of almost 2.5 h [45]. MP-AzeFlu also provided significantly (p = 0.005) greater nasal symptom relief than FP + LOR, which did not differ from placebo during the 4 h study period (p = 0.182). Furthermore, MP-AzeFlu achieved the 1.17 medium MICD in rTNSS at 15 min, compared to both placebo and to FP + LOR. By contrast, patients treated with FP + LOR experienced this difference vs placebo at 210 min, more than 3 h later. When assessing clinical relevance in terms of 50% reduction from baseline in TNSS, 71.3% of patients treated with MP-AzeFlu achieved this clinically relevant response, and did so significantly faster than those treated with FP + LOR (p < 0.001) or placebo (p = 0.002), approximately 2.5 h faster [45]. MP-AzeFlu’s rapid onset of action and rapid clinically relevant effect should improve AR control, with this profile fitting well with prn use of AR medications and rapid treatment switching observed in real life.

## Targeting nasal hyper-reactivity

As many as 36.5% of patients currently on an AR treatment continue to experience poorly controlled AR (i.e. VAS score ≥ 5), with this number remaining essentially the same irrespective of the type of pharmacological treatment used, be it INS (38.5%), oral anti-histamine (34.9%) or multiple therapies (35.9%) [20]. This situation is familiar to HCPs, who consider that 58% of their AR patients have either uncontrolled or poorly controlled disease [39]. Lack of AR control is influenced by 4 factors, namely (1) diagnosis-related (e.g. incorrect diagnosis, presence of co-morbidity); (2) treatment-related (e.g. insufficient efficacy or lack of targeted symptom relief); (3) patient-related (e.g. poor adherence); and (iv) disease-related (e.g. treatment resistant phenotypes) [19]. Using an AR treatment with a rapid onset of action and rapid clinically relevant effect is one way to improve AR control, presumably by addressing patient-related factors; improving adherence to therapy, or perhaps being more forgiving of poor adherence. Another way to achieve better AR control is to address disease-related factors by targeting an alternative disease pathway. Nasal hyperreactivity (NHR) is an important clinical feature of AR, which is present in up to two-thirds of AR patients [46], and may be responsible for breakthrough symptoms frequently reported by AR patients and dissatisfaction with treatment. NHR is defined as an increased sensitivity of the nasal mucosa to environmental, non-specific stimuli (e.g. temperature/humidity changes, smoke, strong odours, exercise and emotional stress), leading to nasal symptoms [47, 48]. The effect of MP-AzeFlu on cold dry air (CDA)-induced-NHR was assessed in a 4-week double-blind, placebo-controlled trial in 28 AR patients with a house dust mite sensitivity [49].

Patients with moderate/severe persistent AR and NHR (i.e. reduced peak nasal inspiratory flow (PNIF) > 20% upon CDA provocation) were randomized 2:1 to receive either MP-AzeFlu (1 spray/nostril bd) or placebo. The effect of MP-AzeFlu on NHR was assessed by PNIF decrease in response to CDA, as well as on the severity of nasal symptoms, measured at baseline and after 7 and 28 days of treatment [49]. Nasal symptom severity was assessed in two ways, by total of 5 symptom scores (T5SS; comprising scores for congestion, nasal itching, rhinorrhoea, sneezing and ocular itching—max score = 15) and by VAS (0–10 cm). AR control was assessed using the AR control test. To investigate the mechanism(s) behind any observed reduction in NHR, the effect of MP-AzeFlu on substance P and β-hexosaminidase concentrations in human nasal secretions was assessed. MP-AzeFlu’s effect on mucosal barrier integrity, mast cell degranulation and airway inflammation in a mouse model of house dust mite-induced NHR, as well as its effect on murine sensory neurons from trigeminal ganglia was also investigated [49].

**Fig. 3:**
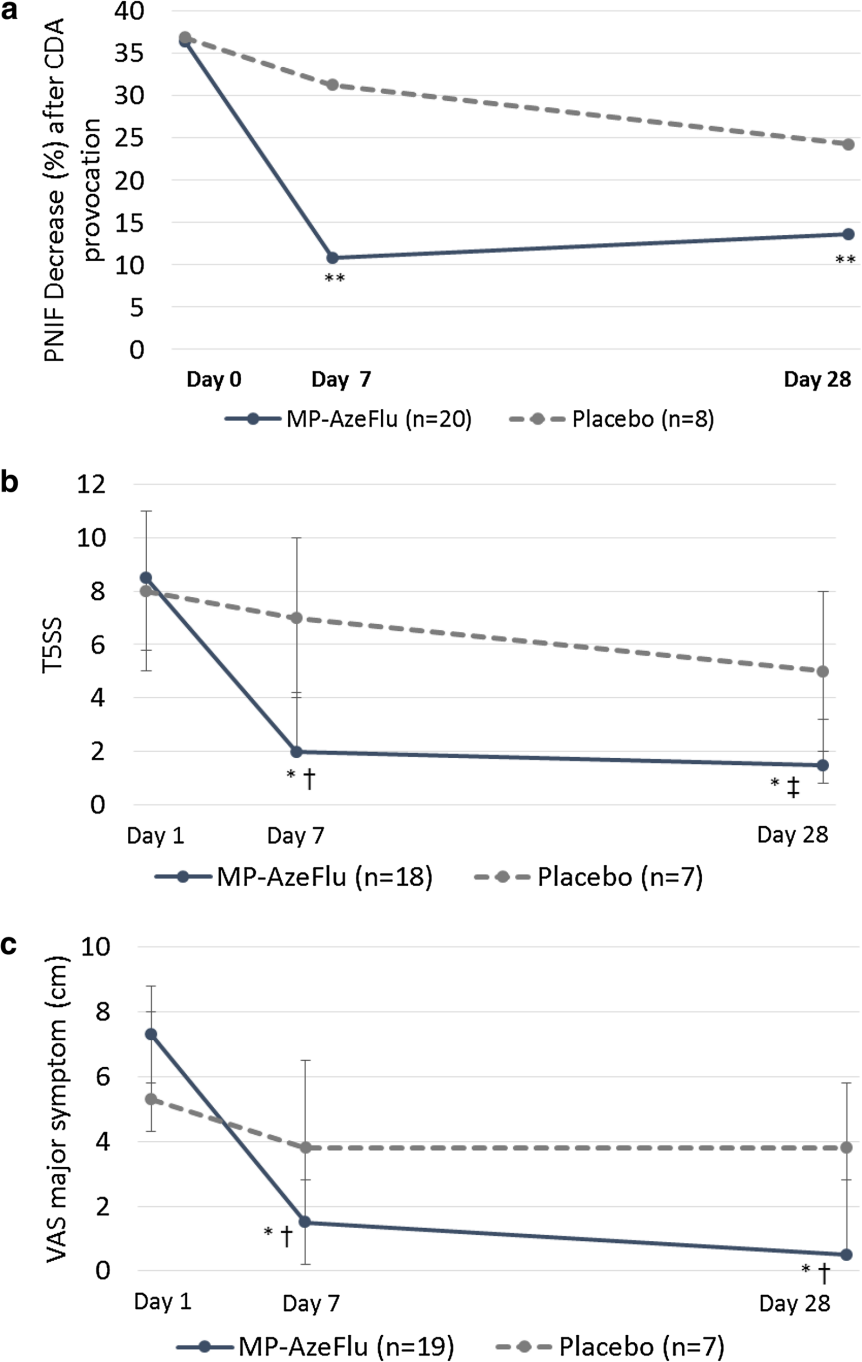
Effect of MP-AzeFlu on nasal-hyperreactivity induced by cold dry air provocation. Nasal hyper-reactivity assessed by **a** PNIF (peak nasal inspiratory flow), **b** T5SS (total of 5 symptom scores) and **c** VAS (visual analogue scale) at day 7 (V1) and day 28 (V2) post-treatment. MP-AzeFlu (MP-azelastine/fluticasone propionate; Dymista^®^; 1 spray/nostril bd; 137 μg/50 μg). CDA: cold dry air. *p < 0.001 vs Day 1; ** p < 0.0001 vs Day 1; † p=0.03 vs placebo; ‡ p=0.003 vs placebo. Modifed from Krohn et al. 2017 [49]

The results showed that MP-AzeFlu significantly (p < 0.0001) reduced NHR (Fig. 3a), with a correlating and significant reduction in nasal symptom severity observed (Fig. 3b, c) [49]. Nasal symptoms were reduced following 28 day’s treatment with MP-AzeFlu by approximately 80% when assessed by T5SS and by 90% when assessed by VAS. Patients treated with MP-AzeFlu for 28 days also experienced significantly (p = 0.035) better AR control compared to placebo. An investigation of the underlying pathophysiology of this NHR reduction revealed that MP-AzeFlu significantly reduced Substance P (p = 0.026) and β-hexosaminidase concentrations (p = 0.036) in nasal secretions, both at Day 7 and Day 28, which was not found in the placebo group [49]. In vitro, AZE + FP inhibited human mast cell degranulation (better than FP and to the same extent as AZE) and decreased murine epithelial permeability (better than AZE and to the same extent as FP). MP-AzeFlu also totally abrogated eosinophils (and neutrophils) in bronchoalveolar lavage fluid in the same murine model of HDM-induced allergic airway inflammation, compared to both AZE and FP, demonstrating a synergistic effect on airway inflammation [49]. Furthermore, only AZE + FP (and not AZE or FP alone) evoked a rapid intracellular Ca^2+^ increase, with repeated applications of AZE + FP resulting in desensitization of sensory neurons expressing the transient receptor potential (TRP) A1 and V1 channels [49]. Activation of these neuronal channels is responsible for the development of idiopathic rhinitis and non-allergic hyperreactivity [50, 51]. MP-AzeFlu’s specific targeting of NHR represents a novel pathway in the pathophysiology of AR, helping to explain the clinical superiority and over-additive effects of MP-AzeFlu seen in clinical practice [52, 53], and providing patients with the complete symptom relief and lack of breakthrough symptoms that they want.

## Discussion

Despite abundant treatment options, AR remains uncontrolled for many patients [39, 54, 55] who are unsurprisingly dissatisfied with their treatment [56]. Dissatisfaction leads to decreased compliance and increased reliance on multiple therapies, including OTC medications [24]. Indeed, 60% of AR patients are “very interested” in finding a new medication, and 25% are “constantly” trying different medications to find one that “works” [24]. AR patients also frequently feel that their HCP does not take their disease seriously and does not understand their personal treatment needs [24]. This lack of effective communication between patients and HCPs most likely contributes to the burden of disease, poor AR control, non-compliance and unhappiness for many patients.

AR control may be significantly improved by improving communication with patients and by prescribing a treatment which takes account of their preferences, expectations and treatment behaviour in real life. MACVIA ARIA has recognized the importance of these issues, by developing apps for AR patients and their physicians to improve communication [42], and by highlighting the importance in the guidelines of patient preference when considering choice of treatment [33]. According to the discrete choice experiment data, AR patients have a strong preference for a treatment which has a rapid onset of action and provides clinically-relevant symptom relief. MP-AzeFlu is the first AR treatment to break the 5 min onset of action threshold [45], much faster than that observed for FP + oral LOR (150 min), or other classes of AR therapy or multiple therapy, measured in the same chamber [57–61]. An AR treatment with a rapid onset of action should encourage AR patients to take it. However, in order for patients to continue taking their AR medication it must also provide rapid clinically-relevant relief. Measured in a RCT setting MP-AzeFlu provided complete symptom relief days faster than an INS [52]. Now, using the more appropriate chamber study design, the onset of clinically relevant symptom relief for MP-AzeFlu is just 15 min, compared to 210 min for FP + LOR [45]. Future AR guidelines should be modified to take account of these new data.

AR control may also be improved in real-life by recognizing that many AR patients also experience NHR [46], and by providing a treatment which targets not only the usual complement of nasal and ocular symptoms associated with AR, but NHR too. The study by Krohn and colleagues [49] is the first to show an effect of MP-AzeFlu on NHR, and to elucidate the cellular pathways underlying this effect. Some of MP-AzeFlu’s novel properties include its ability to reduce mast cell mediators and neuromodulators, and to restore or maintain the mucosal barrier, thus preventing ingress of allergens to the submucosa. Furthermore, AZE and FP worked synergistically together to reduce airway inflammation in vitro and interacted in a unique way to desensitize sensory neurons expressing TRPA1 and TRPV1 [49]. Others have found that treatment with topical mometasone furoate objectively reduced nasal congestion and NHR to histamine in children and adolescents with PAR [62].

## Conclusion

Taken together these data should change the way that AR is managed in real-life by encouraging frank and open communication with patients, as well as prescription of medication which takes patient preferences, disease phenotype and treatment behaviour into account. It’s time to take a more pragmatic approach to AR management in order to provide a personalized medicine, centred around the patient [63]; to recognize that in real-life patients do not behave as they do in RCTs. A pragmatic approach involves fitting the AR treatment to the patient, rather than the patient to the treatment. With the most rapid onset of action and onset of clinically-relevant effect of any AR medication currently available, as well as proven efficacy in the treatment of NHR, MP-AzeFlu is an AR treatment which is tailored to the patient.

## Abbreviations

AR: allergic rhinitis
ARIA: Allergic Rhinitis and its Impact on Asthma
AZE: azelastine
CDA: cold dry air
CDSS: clinical decision support system
EUFOREA: European forum for research and education in allergy and airways diseases
FP: fluticasone propionate
HCP: health care provider
HDM: house dust mite
INS: intranasal corticosteroid
LOR: loratadine
MACVIA: Contre les Maladies Chroniques pour un Vieillissement Actif
MASK: Mobile Airways Sentinel Network
MICD: minimally important clinical difference
NHR: nasal hyper-reactivity
OTC: over the counter
PNIF: peak nasal inspiratory flow
T5SS: total of 5 symptom scores
TNSS: total nasal symptom score
TRP: transient receptor potential
RCT: randomized controlled trials
VAS: visual analogue scale
